# The Structural Relationship between Basic Psychological Needs, Grit, and the Quality of Life of Individuals with Disabilities

**DOI:** 10.3390/ijerph20031758

**Published:** 2023-01-18

**Authors:** Deok-Jin Jang, Chae-Yun Oh, Mun-Gyu Jun, Kyung-Rok Oh, Joon-Hee Lee, Jusun Jang, Sung-Un Park

**Affiliations:** 1 Department of Sports Medicine, Shinhan University, Uijeongbu-si 11644, Gyeonggi-do, Republic of Korea; 2 Department of Coaching, Kyunghee University, Yongin-si 17104, Gyeonggi-do, Republic of Korea; 3 Department of Sports Science, Hanyang University, Ansan-si 15588, Gyeonggi-do, Republic of Korea; 4 Department of Sports and Health, Hwasung Medi-Science University, Hwaseong-si 18274, Gyeonggi-do, Republic of Korea

**Keywords:** individuals with disabilities, basic psychological needs, grit, quality of life

## Abstract

Individuals with disabilities who engage in regular physical activity reduce their risk of diseases such as obesity and heart disease, as well as other risk factors; relieve tense emotions, and improve their quality of life via interaction with others. Despite these advantages, only one out of every four Koreans with a disability engages in physical activity. Grit is the ability to maintain interest and effort towards a goal in the face of adversity and failure. Grit can act as an important factor in increasing the psychological level of individuals with disabilities. We investigated the relationship between basic psychological needs, grit, and the quality of life of disabled individuals to determine if physical activities can improve their quality of life. Our dataset included 296 disabled individuals registered with the Korean Ministry of Health and Welfare. Using structural equation modelling, the direct and indirect effects of grit, quality of life, and psychological needs satisfaction such as competence, relatedness, and autonomy were examined. We found that competence positively affects consistency of interests (β = 0.150, t = 1.854), relatedness positively affects consistency of interests (β = 0.354, t = 4.409), and autonomy has no statistically significant effects (β = 0.101, t = 1.086). Second, competence positively affects perseverance of effort (β = 0.249, t = 3.206), autonomy negatively affects perseverance of effort (β = −0.269, t = −2.880), and relatedness has no statistically significant effects (β = −0.017, t = −0.249). Third, autonomy positively affects quality of life (β = 0.214, t = 2.349) while competence and relatedness had no statistically significant effects (β = −0.018, t = −0.208; β = 0.096, t = 1.288). Fourth, consistency of interests positively affects quality of life (β = 0.312, t = 4.191) while perseverance of effort had no statistically significant effects (β = −0.094, t = −1.480). Fifth, competence was found to have positive indirect effects on quality of life through grit. This study underscores the importance of addressing these three basic psychological needs and elements of grit when designing future quality of life interventions for disabled individuals.

## 1. Introduction

According to the World Health Organization (WHO), there were more than 1 billion individuals with disabilities worldwide as of 2020 [1]. In South Korea, there are about 2.63 million registered individuals with disabilities, constituting 5% of the total population [2]. Although the proportion of individuals with disabilities in Korea is lower than that of the world, according to WHO, the proportion of elderly individuals with disabilities is high in Korea, and 88% of individuals with disabilities live with acquired disorders [3]. Most importantly, the number of people with disabilities has been continuously increasing since 2010 [4].

Adults with disabilities were found to be more likely to develop obesity, heart disease, stroke, diabetes, or cancer than adults without disabilities [5]. Unfortunately, individuals with disabilities report lower levels of physical activity and health-related quality of life than people without disabilities [6]. For individuals with disabilities, engaging in regular physical activity reduces the risk of diseases such as obesity and heart disease, as well as other risk factors [7], relieves tense emotions, and improves the quality of life via interaction with others [8]. Despite these advantages, the physical activity participation rate of individuals with disabilities in Korea was 24.9%, lower than the physical activity participation rate of individuals without disabilities (49.3%) [9]. Participation in regular physical activity can delay aging by strengthening the physical fitness of the disabled, and physical fitness management has a great impact on health and quality of life in old age. Thus, continuous physical activity is an extremely important factor impacting quality of life [10].

In modern society, people with and without disabilities coexist and live together. Owing to their disability, some individuals may show passive dependence on others. This eventually leads to them having negative thoughts about life. Therefore, they experience psychological anxiety due to the physical pain and mental stress caused by their disability. Compared to people without disabilities, individuals with disabilities struggle more often with psychological and mental problems, such as depression and suicidal thoughts [1]. As a result, their quality of life is inevitably reduced.

Self-determination theory (SDT) assumes that it is essential for humans to pursue basic psychological needs (BPNs) such as autonomy, competence, and relatedness [11]. Research has shown that satisfying these three BPNs promotes the development of self-determination motivation, and that it serves as the basis of not only task performance but also psychological well-being [12,13]. SDT also suggests that these three universal psychological needs must be satisfied for individuals to maintain optimum performance and wellbeing [14]. Therefore, the pursuit of BPNs can act as an important psychological variable for individuals with disabilities who experience anxiety along with other mental and physical difficulties.

BPNs are an innate necessity as well as a general human desire. Satisfying these general needs can lead to the development of sound social skills as well as improved internal subjective well-being [15]. BPNs represent a large part of the theory underlying the SDT [12], which demonstrates the relationship between the conceptual meaning of basic human needs and psychological health [16,17]. There are various types of environments and factors that help satisfy the basic needs of individuals that not only provide enjoyment but also promote the autonomous regulation of behavior, thereby ultimately affecting individuals’ performance and adaptation [18]. In addition, through the process of continuing the actions and experiences in a special field that the individual has chosen voluntarily, such as psychological growth and development, one develops an interest, which then becomes passion, and can result in the exhibition of grit [19,20].

Grit is an abbreviation for growth, resilience, intrinsic motivation, and tenacity. It refers to one’s ability to maintain interest and effort toward a goal despite facing adversity and failure [21]. Grit is composed of two factors: consistency of interests (CI) and perseverance of effort (PE) [22]. A person with high grit does not give up even in difficult circumstances, and they instead show a tendency to persist and work toward a goal; by contrast, people with low grit tend to give up easily, such as when experiencing a simple change of heart [23].

Although grit results in success through short-term practice and mastery, it still takes a considerable amount of time for the effect to materialize [19,24]. In addition, Duckworth [19] refers to failure experiences as important in developing grit. According to Lucas, Gratch, Cheng, and Marsella [25], there was no difference in persistence according to the grit level of the group that experienced success. However, in the case of the group that experienced failure, those with a high grit level showed significantly higher persistence. These findings demonstrate that grit develops and is exercised in situations of failure.

Developing grit is challenging, and it may not be out of the ordinary for individuals with a disability to experience more failures than able-bodied individuals in everyday life. Specifically, participating in physical activities can be recognized as essential for individuals with a disability. Furthermore, as the daily life of individuals with a disability consists of various obstacles (big and small) compared to able-bodied individuals, these obstacles can serve as purposeful and as a factor in building grit [26]. In addition, grit can be an important factor for improving the psychological state of individuals with disabilities, and numerous previous studies [27,28,29,30], have verified the relationship between BPNs and grit. However, the current reality is that there is limited research on grit focusing on individuals with disabilities. Therefore, it is necessary to study the influence of grit as a psychological variable that can promote the quality of life of individuals with disabilities.

Individuals with disabilities must often undergo long rehabilitation periods, and after completing rehabilitation, must fully re-enter society. However, there is a vicious cycle in which a person with a disability may feel ashamed about being disabled and therefore unable to fully engage in society, thus causing them to feel further shame and experience more difficulties. Individuals with disabilities who continue forming their identities and participating in social activities involving physical activity show improved psychological stability, reduced depression, and general health promotion. In this context, the rate of physical activity among the disabled in Korea has increased by about 15% since 2010 [31].

Despite showing increased participation in physical activity, individuals with disabilities in Korea often live in limited environments due to their disabilities, and they may receive negative looks from people without disabilities or feel a sense of loss, resulting in a much lower quality of life than people without disabilities [32]. Therefore, this study used structural equation modelling (SEM) to verify the mediating effect of grit on the relationship between BPNs and the quality of life of individuals with disabilities. The following research questions and were addressed in this model: (1) What is the relationship between BPNs, grit, and quality of life? (2) How do BPNs affect quality of life through grit?

## 2. Materials and Methods

### 2.1. Data Collection

The study population in this study was individuals with disabilities who were officially registered with the Ministry of Health and Welfare of South Korea. To achieve the study objective, individuals with disabilities participating in physical activities were also included in the study sample. The survey was conducted from February to September 2020, after obtaining permission from the facility officials where individuals with disabilities participate in physical activities. When distributing the questionnaire, the contents of the questionnaire, purpose of the study, and precautions to be taken when completing the questionnaire were explained in detail to the participants to ensure a valid and reliable process. We requested the participants who were convenience sampled to complete a self-administered questionnaire. Individuals with disabilities who were unable to fill out the questionnaires themselves were assisted by their guardians, and the completed questionnaires were retrieved immediately after completion.

Although SEM does not have an exact standard for sample size, it is recommended that the number of participants should be at least 15 times the number of observed variables to be measured, and it is generally recommended that the sample size be at least 200 [14,33]. Altogether, after discarding 24 unreliable responses, this study analyzed 296/320 sets of data (94.7%), thereby satisfying the minimum number of participants to meet the recommended sample size. Regarding the gender of the participants in this study, 71.3% were men and 28.7% were women. Physical disability was the most common type of disability (60.5%). Preferred forms of physical activity were individual (78%) and group (22%) activities. Physical activity experience was less than one year (5.7%), more than one year and less than two years (6.8%), more than two years and less than three years (8.4%), and more than three years (79.1). Table 1 presents the descriptive statistics of the participants.

### 2.2. Measurement

Validated measures of BPNs in the context of physical activity were used to assess the degree to which participants felt that their needs of autonomy, competence, and relatedness were satisfied [34]. Satisfaction of the psychological need for autonomy (e.g., ‘I am free to participate in any physical activity what I want to do’), relatedness (e.g., ‘I maintain good relationships with other people during physical activity’), and competence (e.g., ‘Physical activity is one of the activities what I am good at’) was measured using 14 items. Grit was measured on a 12-item scale developed by Duckworth et al. [22], which considered two factors: CI (e.g., ‘I focus briefly on an idea or task and then lose interest’) and PE (e.g., ‘Difficulties don’t break me’). To measure quality of life, two items were used based on the study of Min et al. [35], who adapted the WHOQOL-BREF scale developed by the WHO [36], to fit the Korean situation. Quality of life was measured using two questions: ‘How satisfied are you with your quality of life?’ and ‘How satisfied are you with your health condition?’. These items were scored on a 5-point Likert scale ranging from strongly disagree to strongly agree.

### 2.3. Statistical Analysis

We examined the direct and indirect effects of the following variables on physical activity among Korean individuals with disabilities: grit, quality of life, and precursor variables (i.e., the BPNs of competence, relatedness, and autonomy). AMOS 24.0 (IBM Corp., Armonk, NY, USA) was used to conduct the SEM analysis. All variables were continuous, and this study used exploratory factor analysis to examine a measurement model that contains six correlated latent variables (i.e., the BPNs of competence, relatedness, and autonomy; grit of CI and PE; and quality of life). Bootstrapped standard errors were used for the test of indirect effects [37]. The order in which the variables were entered into the model was based on the hypothesized theoretical framework. We used four model fit indices to assess goodness of fit: root mean square error of approximation (RMSEA), chi square, comparative fit index (CFI), and Tucker–Lewis index (TLI).

## 3. Results

### 3.1. Normality of Data and Reliability Analysis

As the SEM assumes multivariate normality, the analysis proceeds under the assumption that the observed variables follow a normal distribution. Therefore, the normality test was used in this study because the parameter estimation method was used as the maximum likelihood method assuming multivariate normality. The normality assessment yielded skewness values ranging from −0.977 to 0.291 and kurtosis values ranging from −0.667 to 1.821, indicating that the data conformed to a normal distribution [38]. In addition, as a result of examining Cronbach’s α to measure the internal consistency of the items measuring constructive concepts, the internal consistency exceeded the standard value of 0.70 suggested by Nunnally and Bernstein [39], thus the items for each factor were reliable. The results of the normality and reliability analyses are shown in Table 2.

### 3.2. The Results of Structural Equation Modeling

This section examines the standardized paths from each latent variable to its respective items. We allowed correlations between all latent variables in the model specification. Maximum likelihood was used as an SEM parameter estimation method. In our measurement models, although the chi-square values were all significant (*p* < 0.05), the RMSEA indexed 0.097, the CFI indexed 0.985, and the TLI indexed 0.925; thus, indicating that our measurement model accurately represented the data [40,41,42].

Table 3 shows the path coefficients of the structural equation models. We got the following results. First, competence positively (+) affects CI (β = 0.150, t = 1.854), relatedness positively (+) affects CI (β = 0.354, t = 4.409), and autonomy has no statistically significant effects (β = 0.101, t = 1.086). Second, competence positively (+) affects PE (β = 0.249, t = 3.206), autonomy negatively (−) affects PE (β = −0.269, t = −2.880), and relatedness has no statistically significant effects (β = −0.017, t = −0.249). Third, autonomy positively (+) affects quality of life (β = 0.214, t = 2.349) while competence and relatedness had no statistically significant effects (β = −0.018, t = −0.208; β = 0.096, t = 1.288). Fourth, CI positively (+) affects quality of life (β = 0.312, t = 4.191) while PE had no statistically significant effects (β = −0.094, t = −1.480).

### 3.3. The Indirect Effect of Grit on the Relationship between BPNs and Quality of Life

Table 4 shows the indirect effect of grit on the relationship between BPNs and quality of life. Competence was found to have positive indirect effect on quality of life through grit. Meanwhile, relatedness and autonomy did not mediate impact on quality of life.

## 4. Discussion and Limitations

The purpose of this study was to identify the structural relationship between basic psychological needs, grit, and quality of life of individuals with disabilities. To achieve this, a survey was administered to 296 individuals with disabilities in South Korea. The prevalence of individuals with disabilities in South Korea is 5.4%, which is significantly lower than the Organization for Economic Co-operation and Development average (15.2%). However, this is because South Korea’s statistics are based on the number of individuals with disabilities registered by the Ministry of Health and Welfare, compared to other countries where the category of individuals with disabilities is relatively broad [43]. In addition, more than 85% of all registered individuals with disabilities are over the age of 40 [3]. Therefore, it can be assumed that the actual number of registered individuals with disabilities in South Korea is probably higher. This study suggests that a physical activity intervention program is needed to re-socialize disabled individuals who are unable to participate in society, thus clarifying the exact population of the disabled. However, despite the importance of physical activity for individuals with disabilities, studies on how to improve quality of life through psychological variables are limited.

Participation in physical activity provides opportunities to understand, consider, and interact with others. Therefore, strengthening competence and relatedness can lead to consistency of interest in life. According to SDT, the experience of satisfying BPNs induces self-determining intrinsic motivation, which allows one to decide which actions to take in a given situation [12]. It is necessary to promote continuous participation in physical activity among individuals with disabilities to help strengthen their autonomy and competence. The increased autonomy and competence achieved through participating in physical activity can also increase one’s perseverance of effort in life.

Regarding the level of physical activity functioning and the participation environment of individuals with disabilities, opportunities for autonomous participation and individual expression of opinion can serve as motivations for continuous participation in physical activity. Although the participation rate in physical activity among individuals with disabilities is slightly increasing in Korea [31], the reality is that there are not enough sports facilities that can be used freely by individuals with disabilities. Similar to the decrease in physical activity for individuals with disabilities, the use of medical institutions decreased by 2.4 days for men with disabilities (0.84 days for non-disabled individuals) and 2.19 days for women with disabilities (0.85 days for able-bodied individuals) [44]. In addition, it was found that the frequency of using medical services was further reduced compared to able-bodied individuals, and in the case of medical expenses, it was found that there was an effect of saving about 215,300 Korean won per person [44]. Therefore, based on policies that can reduce these socioeconomic costs, the quality of life of individuals with disabilities can be improved by expanding the public sports facilities for them to use voluntarily.

Research has shown that students with a high grit level had higher life satisfaction and psychological well-being than students with a low grit level [45]. Without effort and enthusiasm, it is difficult for individuals with disabilities to overcome long-term rehabilitation periods as well as any limitations in movement or communication. The Korean government’s policy to cope with the COVID-19 pandemic, which focuses on non-disabled individuals, exemplifies the fear of infection and alienation experienced by individuals with disabilities, who are more vulnerable to infection. The COVID-19 pandemic is clearly putting increasing restrictions on the physical activities of individuals with disabilities. As a result of the difficulties faced by individuals with disabilities in daily life, navigating one’s life can become a major goal for individuals with disabilities. Consistency of interests—which involves setting goals and not giving up on them—is an essential element in improving one’s quality of life. Although competence does not directly affect quality of life, it is a meaningful result that can improve the quality of life when grit is mediated.

The interpretation of these results is subject to several limitations. First, all data were self-reported, which might have resulted in respondent, recall, and/or interviewer biases. A sense of psychological atrophy caused by physical alienation among individuals with disabilities, along with face-saving behavior, might have led the respondents to under-report their perceptions and behaviors. Second, owing to the cross-sectional design of this study, causal relationships among the variables could not be determined. Third, face-to-face investigations were extremely limited due to the COVID-19 pandemic, as individuals with disabilities are a vulnerable group. In addition, from a practical perspective, it was difficult to secure enough cases for each type of disability due to major differences between disabilities. Therefore, this study’s results are limited in their examination of differences by disability type. Fourth, the results may not be generalizable to other populations because we focused exclusively on individuals with disabilities who participated in physical activity in Korea.

## 5. Conclusions

Despite these limitations, the results of this study contribute to the literature by providing valuable information on the need to consider psychological variables that can improve the quality of life of individuals with disabilities. Our findings are important because individuals with disabilities in Korea are largely socially marginalized in terms of their participation in physical activity. Such individuals, regardless of their autonomy and relatedness, can be subject to societal prejudices and views that can prevent them from participating in physical activity. Our results also suggest that producing autonomy and competence-mediated grit, along with providing a supportive social environment, are important for improving the quality of life of individuals with disabilities. This study highlights the importance of addressing grit and satisfaction of psychological needs when attempting to improve the quality of life for this underrepresented group.

## Figures and Tables

**Table 1:** Socio-demographic characteristics of the participants (*n* = 296).

Characteristics		*n*	%
Gender	Male	211	71.3
	Female	85	28.7
Type of disability	Physical disability	179	60.5
	Brain lesions	57	19.3
	Hearing impairment	33	11.1
	Visual impairment	20	6.8
	Others	7	2.4
Physical activity experience	Less than one year	17	5.7
	More than one year and less than two years	20	6.8
	More than two years and less than three years	25	8.4
	More than three years	234	79.1
Preferred type of physical activity	Individual	231	78
	Group	65	22

**Table 2:** The results of normality and reliability analyses.

	Competence	Relatedness	Autonomy	Consistency of Interests	Perseverance of Effort	Quality of Life
Mean	4.301	4.056	3.969	2.701	3.839	3.604
Standard deviation	0.668	0.793	0.769	0.862	0.735	0.842
Skewness	−0.977	−0.706	−0.787	0.291	−0.163	0.001
Kurtosis	1.821	0.274	1.004	0.266	−0.598	−0.667
Cronbach α	0.899	0.900	0.842	0.855	0.861	0.739

**Table 3:** Result for structural equation modeling.

Items	Path	ß	SE	C.R
1-1	Competence -> Consistency of interests	0.150	0.105	1.854 **
1-2	Relatedness -> Consistency of interests	0.354	0.073	4.409 ***
1-3	Autonomy -> Consistency of interests	0.101	0.101	1.086
2-1	Competence -> Perseverance of effort	0.249	0.074	3.206 **
2-2	Relatedness -> Perseverance of effort	−0.017	0.077	−0.249
2-3	Autonomy -> Perseverance of effort	−0.269	0.100	−2.880 **
3-1	Competence -> Quality of life	−0.018	0.096	−0.208
3-2	Relatedness -> Quality of life	0.096	0.098	1.288
3-3	Autonomy -> Quality of life	0.214	0.099	2.349 *
4-1	Consistency of interests -> Quality of life	0.312	0.088	4.191 ***
4-2	Perseverance of effort -> Quality of life	−0.094	0.065	−1.480
x^2^ = 8.753, df = 3, RMSEA = 0.097, CFI = 0.985, TLI = 0.925				

*** *p* < 0.001, ** *p* < 0.01, * *p* < 0.05, ß = standard coefficient, SE = standard error; C.R = critical ratio, x^2^ = chi square, df = degrees of freedom, RMSEA = root mean square error of approximation, CFI = comparative fit index, TLI = Tucker–Lewis index.

**Table 4:** The indirect effect of grit on the relationship between BPNs and quality of life.

Indirect Effects				Coefficients
Competence	Grit	Quality of life	Physical activity	0.068 **
Relatedness				−0.019
Autonomy				0.136

** *p* < 0.01, BPNs = basic psychological needs.

## Data Availability

Not applicable.
